# A screening model for advanced colorectal neoplasia based on tumor markers and inflammatory indices: a retrospective study with an online risk calculator

**DOI:** 10.3389/fonc.2026.1841258

**Published:** 2026-06-22

**Authors:** Chang Zhang, Lei Jin, Shihao Wu, Liang Lu, Shui Jin

**Affiliations:** 1 Department of Gastroenterology, The Fourth Affiliated Hospital of Anhui Medical University, Hefei, China; 2 Department of Burns, The First Affiliated Hospital of Anhui Medical University, Hefei, China

**Keywords:** advanced colorectal neoplasia, inflammation biomarkers, machine learning, online risk calculator, SHAP

## Abstract

**Objectives:**

The goal is to design and validate a noninvasive screening model for advanced colorectal neoplasia(ACN) utilizing routine blood tumor markers and inflammatory indices, and to develop an online calculator for assessing individual risk.

**Methods:**

In this retrospective analysis, 1,290 patients who underwent colonoscopy and had full preoperative blood test results were included. Based on pathology, patients were categorized into a group with advanced colorectal neoplasms and another with non-advanced neoplasms. Patients were randomly assigned to a training set, making up 70%, and a test set, making up 30%.Candidate variables were initially screened using univariate logistic regression, and those with statistical significance were then included in a multivariate logistic regression model to determine independent predictors. To assess potential nonlinear relationships between continuous variables and the risk of advanced neoplasms, restricted cubic splines were employed. Prediction models were constructed using five machine learning algorithms and evaluated using the area under the receiver operating characteristic curve. The robustness of key predictors was further assessed through sensitivity and stratified analyses. SHAP was applied to interpret the final model, which was subsequently implemented as an online calculator.

**Results:**

Among the 1,290 patients, 210 were diagnosed with advanced colorectal neoplasms. CEA, SII, NLR, ALB, and PLR were identified as independent predictors, and their associations remained stable across sensitivity and stratified analyses. Among the models, XGBoost achieved the best performance, with an AUC of 0.956 (95% CI 0.936–0.976). SHAP analysis identified SII as the most influential predictor.

**Conclusion:**

A machine learning model based on key blood markers such as CEA, SII, and NLR can effectively support noninvasive screening for advanced colorectal neoplasms. This online calculator is a convenient and practical resource for evaluating risk on an individual basis in clinical practice.

## 1 Introduction

Globally, colorectal cancer (CRC) is still a major contributor to cancer-related sickness and fatalities (1, 2). It usually progresses according to the recognized adenoma–carcinoma sequence (3, 4). During this process, advanced colorectal neoplasia includes both advanced adenomas and early-stage cancer. Advanced adenomas are characterized by lesions that are 10 mm or larger, show high-grade intraepithelial neoplasia, or have a villous or tubulovillous structure (5–7). This stage represents a critical and potentially reversible phase in the progression of CRC.

Early detection and prompt intervention are vital approaches to prevent invasive cancer progression and cut down on CRC-related mortality (8). Early detection of lesions allows for effective treatment before they advance into a more aggressive disease, improving patient outcomes and reducing the risk of death associated with CRC. Therefore, there is an increasing need for simple, noninvasive, and cost-effective approaches to identify individuals at high risk for ACN.

Currently, CRC screening mainly relies on colonoscopy and stool-based tests (9–14). When it comes to identifying colorectal lesions, colonoscopy is considered the top standard (15, 16). Nevertheless, its invasive nature, high expense, and relatively low patient adherence restrict its broad use in population-wide screening initiatives (17). Tests using stool samples, such as fecal occult blood tests and stool DNA tests, offer noninvasive options. Nevertheless, their performance remains suboptimal for detecting precancerous lesions due to limited sensitivity and specificity (18, 19). These limitations highlight the need for alternative screening strategies that are more acceptable and feasible for large-scale implementation.

In recent years, tumor markers and inflammation-related blood parameters have attracted increasing attention in CRC research. There is a strong connection between these biomarkers and the initiation, development, and clinical outcomes of tumors. Studies show that carcinoembryonic antigen (CEA) and inflammation-related ratios, including the neutrophil-to-lymphocyte ratio (NLR) and platelet-to-lymphocyte ratio (PLR), are closely connected to tumor growth and advancement (20–22). Recent multicenter findings have further confirmed that integrating inflammatory and nutritional biomarkers provides robust prognostic value in colorectal cancer. In stage I–III colorectal cancer, composite indices such as the neutrophil-to-albumin ratio (NPAR) and albumin-to-globulin ratio (AGR) have been shown to independently predict outcomes (23). Similarly, the platelet-to-albumin ratio (PAR) and the cancer inflammation prognostic index (CIPI) have demonstrated strong predictive ability for postoperative survival (24). In addition, a novel immune-nutritional prognostic ratio (INPR), which integrates the prognostic immune nutritional index (PINI) and the lymphocyte-to-monocyte ratio (LMR), has been reported to outperform single biomarkers in risk stratification (25). Collectively, these results emphasize the critical role of the systemic immune–inflammatory–nutritional axis in colorectal tumor development and support the rationale for integrating multiple blood-based biomarkers—including inflammatory indices and nutritional indicators—into predictive models for advanced colorectal neoplasia. Importantly, although these composite indices differ in formulation, they fundamentally reflect the interplay between inflammation, immune status, and nutritional condition, which are closely linked to tumor development. However, existing studies have several limitations. Most have focused on single or a limited number of biomarkers (26), which may not adequately capture the complex biological processes underlying tumorigenesis. Most existing studies have primarily focused on established CRC rather than ACN, which represents a critical and potentially reversible stage within the adenoma–carcinoma sequence. From a clinical perspective, early identification and intervention at this stage may effectively prevent progression to invasive cancer. In addition, traditional regression models may be insufficient to fully capture the complex nonlinear relationships and interactions among hematological variables, thereby limiting their predictive performance.

With the rapid development of medical big data, clinical prediction models—particularly those based on machine learning—have become a major focus of research. Machine learning algorithms are more adept than traditional statistical methods at managing high-dimensional data, intricate interactions, and nonlinear relationships (27–29). Given the multifactorial nature of hematological indicators, machine learning may provide a more effective framework for risk prediction. However, in the field of ACN screening, machine learning models based on blood-derived markers remain limited, and the issue of model interpretability has not been sufficiently addressed.

Based on these considerations, the present study aims to integrate tumor markers with inflammation-related hematological indicators to develop a predictive model for advanced colorectal neoplasms. Several machine learning algorithms were developed and evaluated to determine the most effective model. We implemented Shapley Additive Explanations (SHAP) to quantify each predictor’s contribution, enhancing interpretability. Finally, we developed an online risk calculator to provide a simple and interpretable tool for individualized risk assessment. Employing this technique could lead to earlier identification of high-risk individuals and make CRC screening strategies more efficient.

## 2 Materials and methods

### 2.1 Research participants and structure

From January 2020 to January 2025, 1,290 patients who had colonoscopies at the Digestive Endoscopy Center of the Fourth Affiliated Hospital of Anhui Medical University were retrospectively and consecutively enrolled. All patients completed a full set of venous blood tests within one week before the procedure. To maintain the model’s stability, we checked if the sample size was sufficient according to the events per variable guideline.(EPV). It is generally recommended that each predictive variable should correspond to at least 10 events (30). In this study, there were 210 cases of advanced colorectal neoplasms. Five variables were ultimately included in the model, and the EPV of 42 fulfilled the necessary conditions for developing the model.

Inclusion criteria: 1) Age ≥ 18 years; 2) Completion of a full colonoscopy with a clear pathological diagnosis; 3) Availability of complete preoperative blood test results.

Exclusion criteria: 1) Previous occurrence of colorectal cancer or other malignant neoplasms; 2) Receipt of chemotherapy, radiotherapy, immunotherapy, or targeted therapy before examination; 3) Presence of acute infection, active inflammatory bowel disease, hematologic disorders, or severe liver or kidney dysfunction; 4) Incomplete clinical or follow-up data.

The pathology results were used to classify enrolled patients into advanced and non-advanced groups. Histopathology confirmed that ACN involves either advanced adenoma or early-stage CRC. Advanced adenoma was defined as an adenoma meeting at least one of the following criteria: (1) size ≥10 mm; (2) high-grade intraepithelial neoplasia; or (3) villous or tubulovillous histology with at least 25% villous component. Early-stage colorectal cancer was defined as invasive adenocarcinoma confined to the submucosa, classified as pT1, without evidence of distant metastasis. The non-advanced group included patients with (1) normal mucosa; (2) non-neoplastic polyps such as hyperplastic or inflammatory polyps; or (3) low-grade adenomas, defined as tubular adenomas with low-grade dysplasia and a size <10 mm. All pathological diagnoses were independently assessed by two expert gastrointestinal pathologists who did not have access to clinical and laboratory data. In instances of differing opinions, a third senior pathologist was consulted to reach an agreement. Group classification was based solely on pathological findings and was performed independently of the laboratory variables used in model development.

### 2.2 Data collection and laboratory parameters

Baseline demographic information were collected for all enrolled patients. Peripheral venous blood was drawn after overnight fasting within 7 days prior to colonoscopy. Routine laboratory examinations were then carried out. All laboratory variables were measured using standardized automated analyzers following institutional quality control procedures.

#### 2.2.1 Routine laboratory parameters

(1) Blood routine tests: neutrophil count, lymphocyte count, monocyte count, platelet count, red blood cell count, hemoglobin, hematocrit, and red blood cell distribution width. (2) Liver function: alanine aminotransferase (ALT), aspartate aminotransferase (AST), total protein (TP), albumin (ALB), and albumin/globulin ratio (A/G). (3) Renal function: urea and creatinine. (4) Other markers: serum calcium, fibrinogen, and prealbumin.

#### 2.2.2 Tumor markers

Carcinoembryonic antigen (CEA), alpha-fetoprotein (AFP), carbohydrate antigen 19-9 (CA199), carbohydrate antigen 125 (CA125), carbohydrate antigen 153 (CA153), and carbohydrate antigen 724 (CA724).

#### 2.2.3 Inflammation- and nutrition-related indices

Based on the above routine parameters, the following ratios and indices were calculated: Neutrophil-to-lymphocyte ratio (NLR) = neutrophil count/lymphocyte count;Platelet-to-lymphocyte ratio (PLR) = platelet count/lymphocyte count;Lymphocyte-to-monocyte ratio (LMR) = lymphocyte count/monocyte count;Monocyte-to-lymphocyte ratio (MLR) = monocyte count/lymphocyte count;Platelet-to-neutrophil ratio (PNR) = platelet count/neutrophil count;Hemoglobin-to-red cell distribution width ratio (HRR) = hemoglobin/red blood cell distribution width;Hemoglobin-to-platelet ratio (HPR) = hemoglobin/platelet count;Fibrinogen-to-prealbumin ratio (FPR) = fibrinogen/prealbumin;Systemic immune-inflammation index (SII) = platelet count × neutrophil count/lymphocyte count;Prognostic nutritional index (PNI) = albumin (g/L) + 5 × lymphocyte count (10^9^/L);All the above variables were included as potential predictors in the subsequent variable selection and model building process.

### 2.3 Statistical analysis and model development

Statistical analyses were performed using SPSS 26.0 and R. Missing data (<5%) were handled using multiple imputation by chained equations (MICE) with the “mice” package, generating five datasets over 10 iterations. Continuous variables were imputed using predictive mean matching, and categorical variables using logistic regression. Missing data proportions are shown in **Supplementary Table S1**, and results were pooled according to Rubin’s rules. Continuous variables are reported as median (IQR) and compared using the Mann–Whitney U test, while categorical variables are expressed as counts (percentages) and compared using the chi-square or Fisher’s exact test. Advanced colorectal neoplasia was defined as the outcome. Univariate logistic regression was used to screen predictors, and variables with P < 0.05 were entered into a multivariable model to identify independent factors. Odds ratios (ORs) with 95% confidence intervals (CIs) were reported. Variables such as age and renal function were considered potential confounders and were further evaluated in robustness analyses rather than being included in the final model. Sensitivity analyses were conducted using stepwise adjustment models with sequential inclusion of key clinical variables (age, liver function, renal function, ALB, and Hb). Stratified analyses were also performed, and interaction effects were assessed using likelihood ratio tests.

### 2.4 Model development and validation

For model development, the dataset was randomly divided into a training set (70%) and a test set (30%). Prior to model training, continuous variables were standardized using z-score normalization. Five machine learning algorithms were applied to construct prediction models, including gradient boosting machine (GBM), random forest (RF), extreme gradient boosting (XGBoost), classification and regression tree (CART), and logistic regression (LR). Hyperparameter tuning for all machine learning models was conducted using repeated 10-fold cross-validation (10 repeats) combined with a grid search strategy. For logistic regression, default parameters (glm) were used. For the CART model, the complexity parameter (cp) was optimized to control tree pruning. For the random forest model, the number of variables randomly selected at each split (mtry) was tuned, while the number of trees was fixed at 500. For the GBM model, key parameters—including interaction depth, learning rate (shrinkage), and the number of trees (n.trees)—were optimized to balance model complexity and predictive performance. For the XGBoost model, multiple hyperparameters were optimized, including the number of boosting rounds (nrounds), maximum tree depth (max_depth), learning rate (eta), subsample ratio (subsample), column sampling ratio (colsample_bytree), and minimum loss reduction (gamma). In addition, L1 (alpha) and L2 (lambda) regularization parameters were tuned. Early stopping based on validation AUC (with 10 rounds of patience) was applied to prevent overfitting.

Model performance was evaluated from multiple perspectives. Discrimination was measured using the area under the receiver operating characteristic curve (AUC), while additional metrics included accuracy, sensitivity, specificity, the Kappa statistic, and the Matthews correlation coefficient. Calibration was assessed using the Brier score, with calibration curves further illustrating the agreement between predicted and observed risks (**Supplementary Figure S1**). Clinical utility was evaluated using decision curve analysis. The final model was selected based on overall performance across these metrics. SHAP analysis was applied to quantify feature contributions and improve model interpretability. An online risk calculator was subsequently developed to support clinical application.

## 3 Results

### 3.1 Study flowchart

**Figure 1:**
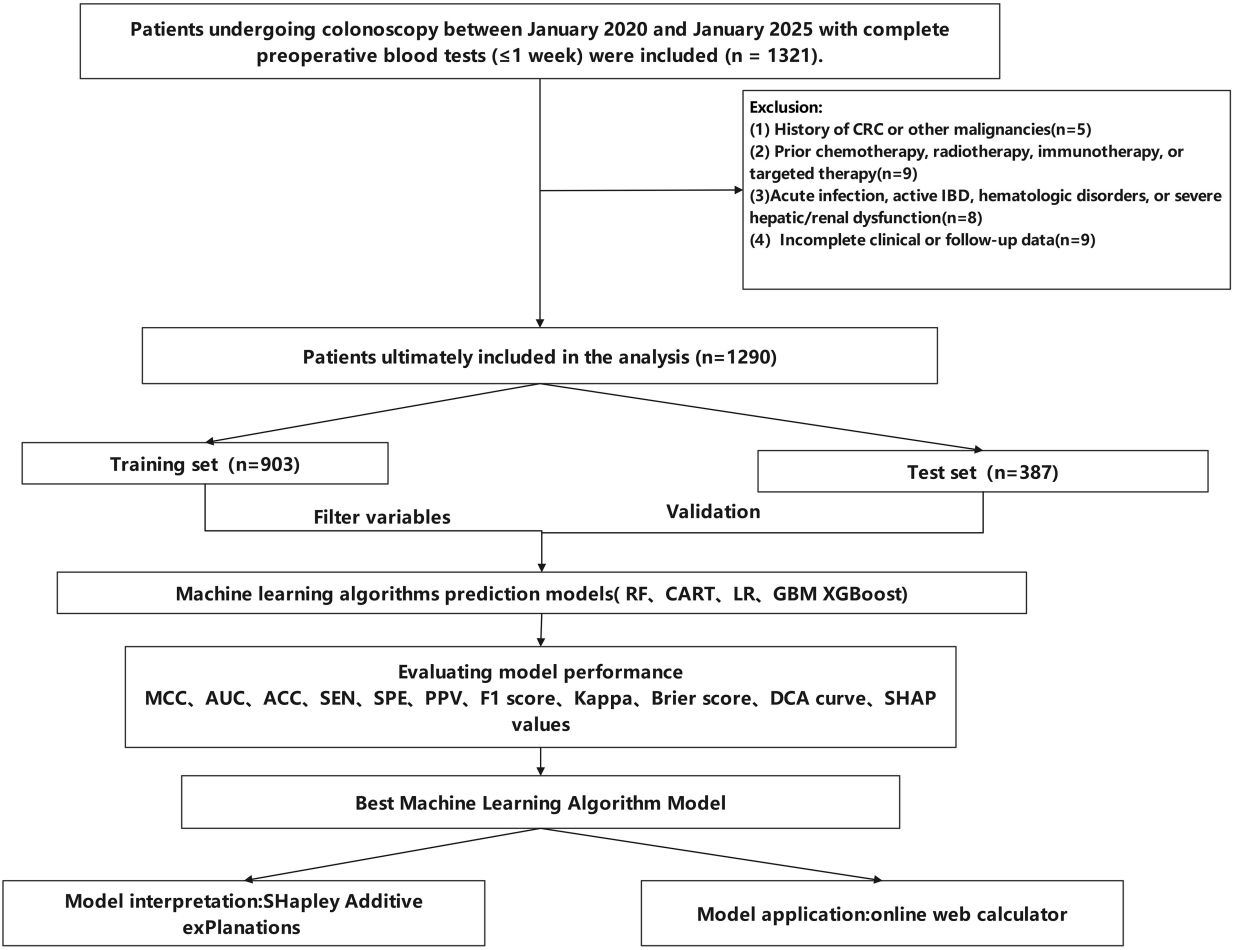
Flowchart of patient selection and study design. A total of 1,321 patients who underwent colonoscopy with complete preoperative blood tests were initially screened. After applying the exclusion criteria, 1,290 patients were included in the final analysis. The dataset was randomly divided into a training set (n = 903) and a test set (n = 387). Variable selection, model development using machine learning algorithms, performance evaluation, and model interpretation using SHAP were subsequently performed.

Following the application of inclusion and exclusion criteria, 1,290 patients were enrolled. Among them, 210 individuals (16.3%) were found to have advanced colorectal neoplasia, whereas 1,080 (83.7%) had non-advanced lesions. The flow of patient enrollment and analysis is presented in **Figure 1**. In a 7:3 ratio, the dataset was randomly divided into a training cohort consisting of 903 samples and a test cohort consisting of 387 samples. Baseline variables were comparable between the two groups (**Supplementary Table S2**). In total, 28 potential predictors were obtained for each subject. **Table 1** summarizes the comprehensive clinical features of the training cohort.

**Table 1:** Clinical characteristics of patients in the training cohort (n=903).

Variables	Non-advanced group(n=756)	Advanced group (147)	P-value
Age [M(Q1-Q3),years]	58.00 [52.00, 67.00]	67.00 [56.00, 71.00]	<0.001
Ca [M(Q1-Q3),mmol/L]	2.33 [2.00, 2.42]	2.32 [2.05, 2.45]	0.471
Urea [M(Q1-Q3),mmol/L]	5.70 [4.80, 6.70]	5.70 [4.90, 7.20]	0.243
Creatinine [M(Q1-Q3),μmol/L]	69.00 [58.00, 83.00]	67.90 [57.00, 82.10]	0.222
FIB [M(Q1-Q3),g/L]	2.74 [2.37, 3.14]	2.85 [2.29, 3.48]	0.317
AFP [M(Q1-Q3),ng/mL]	2.44 [1.82, 3.30]	2.52 [1.61, 3.54]	0.955
CEA [M(Q1-Q3),ng/mL]	2.34 [1.60, 3.86]	11.00 [2.36, 13.00]	<0.001
CA199 [M(Q1-Q3),U/mL]	4.01 [2.00, 7.43]	19.00 [9.00, 39.00]	<0.001
CA125 [M(Q1-Q3),U/mL]	10.90 [8.17, 15.20]	14.00 [10.55, 21.00]	<0.001
CA153 [M(Q1-Q3),U/mL]	8.00 [6.27, 11.60]	9.50 [6.50, 13.30]	0.037
CA724 [M(Q1-Q3),U/mL]	1.82 [1.05, 3.09]	1.88 [0.50, 3.83]	0.799
ALT [M(Q1-Q3),U/L]	21.00 [16.00, 31.00]	24.00 [15.00, 36.00]	0.779
AST [M(Q1-Q3),U/L]	23.00 [19.00, 28.00]	25.00 [16.50, 36.00]	0.112
TP [M(Q1-Q3),g/L]	71.60 [66.97, 75.30]	67.30 [61.00, 71.25]	<0.001
ALB [M(Q1-Q3),g/L]	45.20 [41.40, 47.70]	28.00 [24.00, 41.40]	<0.001
A/G	1.72 [1.51, 1.89]	1.54 [1.27, 1.95]	0.003
RBC [M(Q1-Q3),×10¹²/L]	4.61 [4.25, 4.98]	4.13 [3.71, 4.96]	<0.001
Hb [M(Q1-Q3),g/L]	134.00 [115.00, 145.25]	128.00 [112.00, 142.00]	0.089
MCHC [M(Q1-Q3),g/L]	333.00 [326.00, 340.00]	335.00 [326.50, 344.00]	0.191
NLR [M(Q1-Q3)]	2.00 [1.56, 2.82]	5.00 [3.00, 5.74]	<0.001
PLR [M(Q1-Q3)]	114.10 [86.00, 149.18]	159.05 [104.19, 222.00]	<0.001
PNI [M(Q1-Q3)]	54.25 [50.58, 58.16]	35.75 [31.32, 51.98]	<0.001
SII [M(Q1-Q3)]	327.55 [200.00, 488.23]	634.00 [341.94, 777.00]	<0.001
FPR [M(Q1-Q3)]	15.00 [12.00, 20.00]	31.00 [23.00, 36.00]	<0.001
HRR [M(Q1-Q3)]	9.00 [8.00, 10.49]	11.43 [9.48, 13.00]	<0.001
HPR [M(Q1-Q3)]	0.75 [0.62, 0.91]	0.77 [0.54, 1.01]	0.882
LMR [M(Q1-Q3)]	4.51 [3.08, 5.92]	3.19 [2.02, 4.88]	<0.001
MLR [M(Q1-Q3)]	0.20 [0.16, 0.26]	0.23 [0.17, 0.32]	0.028

### 3.2 Feature selection

**Figure 2:**
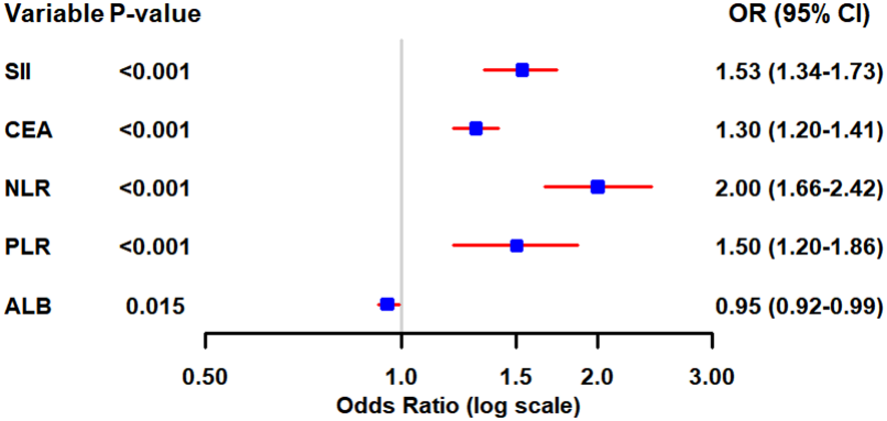
Multivariable logistic regression analysis of independent predictors for ACN. Forest plot showing ORs with 95% CIs for each independent predictor identified in the multivariable model.

Univariate logistic regression identified several clinical and laboratory factors related to advanced colorectal neoplasia (P < 0.05). Significant variables included CEA, CA199, PNI, SII, NLR, PLR, age, CA125, creatinine, AST, TP, ALB, A/G, RBC, FPR. HRR. LMR and MLR. Variables with statistical significance were entered into the multivariable logistic model. The analysis showed that SII [P < 0.001, OR (95% CI) = 1.53 (1.34–1.73)], CEA [P < 0.001, OR (95% CI) = 1.30 (1.20–1.41)], NLR [P < 0.001, OR (95% CI) = 2.00 (1.66–2.42)], PLR [P < 0.001, OR (95% CI) = 1.50 (1.20–1.86)], and ALB [P = 0.015, OR (95% CI) = 0.95 (0.92–0.99)] remained independently associated with advanced colorectal tumors, as presented in **Figure 2**.

### 3.3 Relationship analysis among selected variables

**Figure 3:**
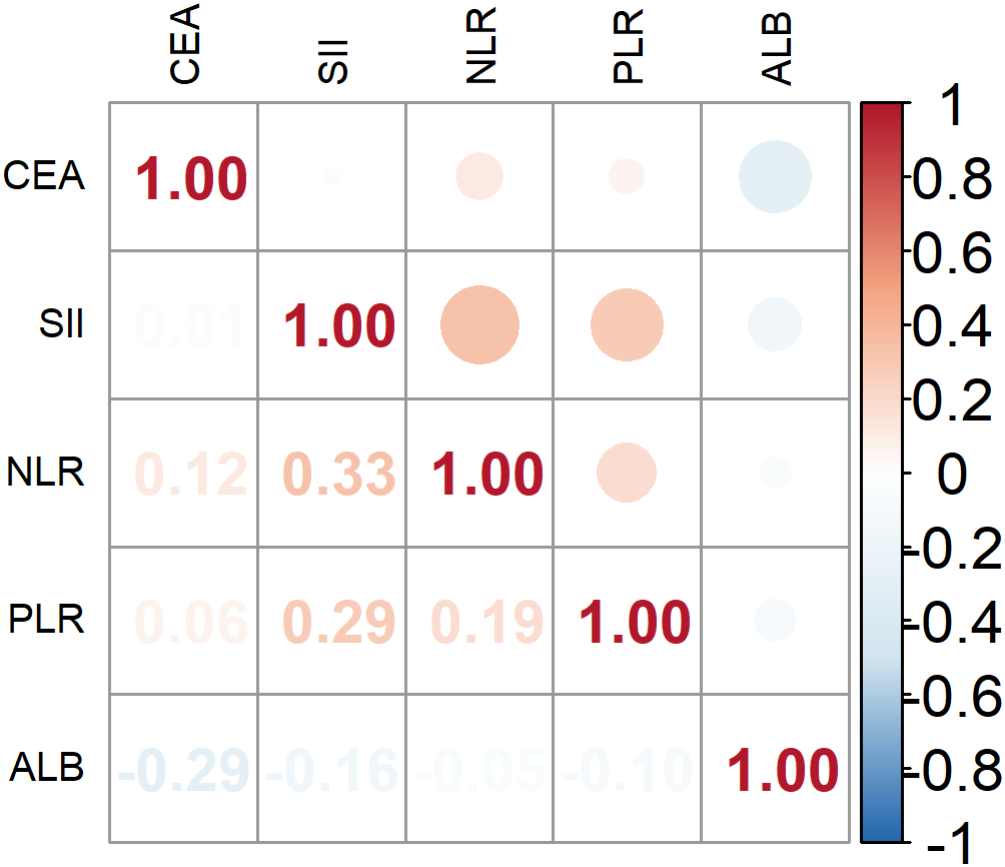
Correlation heatmap of selected predictors. The image presents pairwise Spearman correlations among CEA, SII, NLR, PLR, and ALB. Color intensity and circle size represent the strength and direction of correlations (red: positive; blue: negative). Overall, correlations between variables were weak to moderate (all |r| < 0.4), indicating minimal multicollinearity and suggesting that each predictor contributes relatively independent information to the model. From a clinical perspective, these biomarkers reflect distinct biological domains: CEA represents tumor burden, SII, NLR, and PLR reflect systemic inflammatory status, and ALB indicates nutritional condition. The limited intercorrelation supports their complementary roles and justifies their combined use in improving risk prediction for advanced colorectal neoplasia.

**Figure 4:**
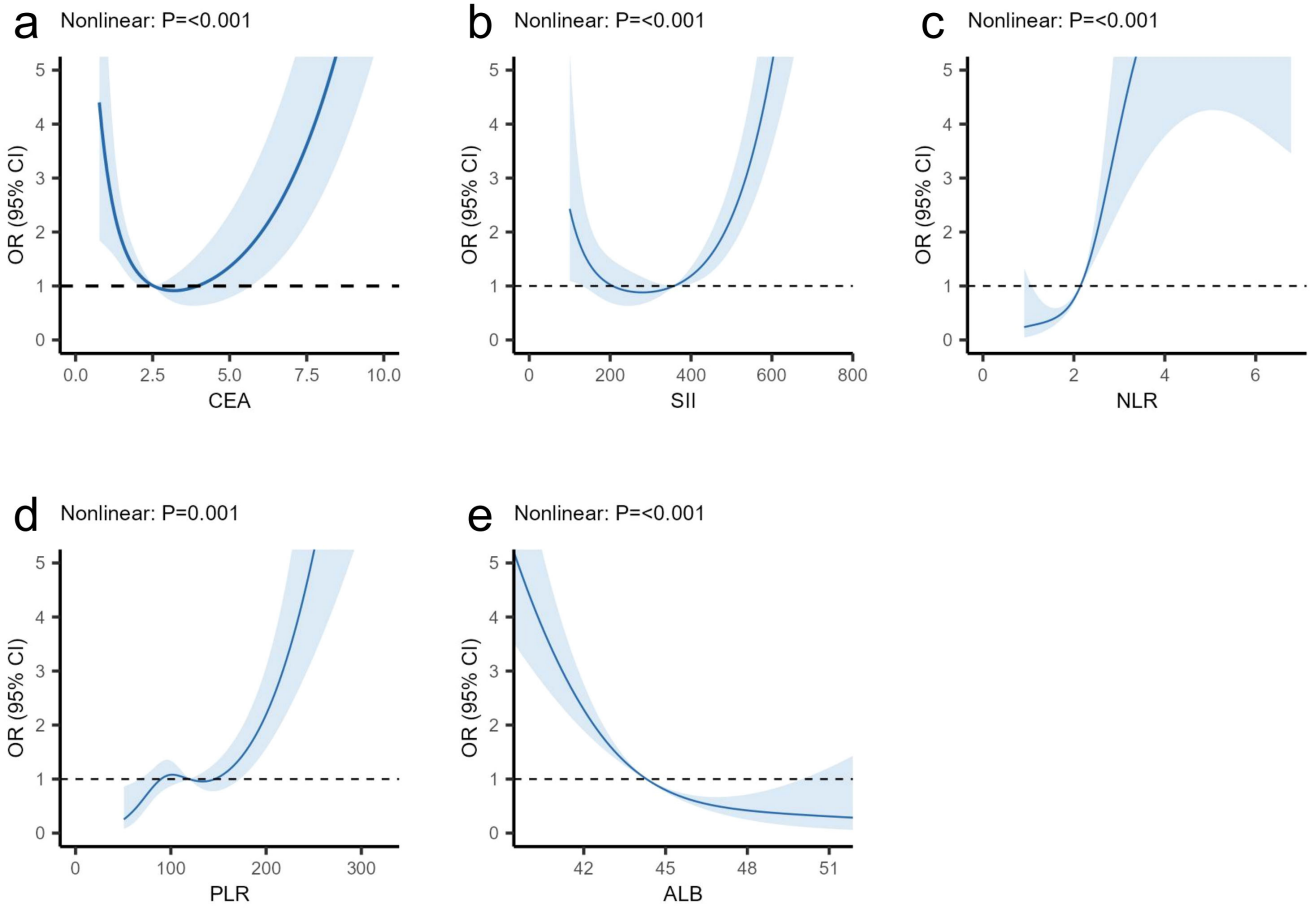
Restricted cubic spline (RCS) analysis of continuous predictors. **(a)** CEA; **(b)** SII; **(c)** NLR; **(d)** PLR; **(e)** ALB. The x-axis represents the value of each variable, and the y-axis represents the OR with 95% CIs for advanced colorectal neoplasia. The dashed horizontal line indicates OR = 1. The P value for nonlinearity is shown in each panel, with P < 0.05 indicating a significant nonlinear association.

We then assessed the relationships among the five selected predictors using correlation analysis, and a heatmap was plotted (**Figure 3**). All correlation coefficients were less than 0.4, indicating no significant multicollinearity among the predictors. To evaluate possible nonlinear interactions between these factors and the outcome, restricted cubic spline(RCS) models were applied. After adjustment for confounders, the RCS curves indicated nonlinear associations for CEA, SII, NLR, PLR, and ALB (**Figure 4**).These variables were subsequently entered into five machine learning methods to develop prediction models.

### 3.4 Robustness analysis of key predictors

When interpreting inflammation-based biomarkers, it is essential to account for the potential influence of confounding and effect modification. In the present study, SII was selected as a representative inflammation-related biomarker for robustness evaluation, given that it integrates neutrophils, lymphocytes, and platelets into a composite index reflecting systemic inflammatory and immune status. We therefore conducted both stepwise sensitivity analyses and stratified analyses to comprehensively assess its robustness. The stepwise adjustment models (**Table 2**) indicated that the link between SII and advanced colorectal neoplasia stayed stable after sequential adjustment for age, liver function, renal function, nutritional status, and hematological parameters. In the fully adjusted model, the effect size was only slightly attenuated, with the odds ratio decreasing from 1.56 to 1.46, supporting the robustness of SII and indicating that its predictive value is not substantially driven by major confounding factors. Similar patterns were observed for CEA (**Supplementary Table S3**), further supporting the consistency of both inflammation-related and tumor-related predictors across different adjustment models.

**Table 2:** Sensitivity analysis for the association between SII and advanced colorectal neoplasia with sequential adjustment for potential confounders.

Model	Adjustments	OR (95% CI)	P value
Model A	None	1.56 (1.45–1.69)	<0.001
Model B	+ Age	1.55 (1.43–1.67)	<0.001
Model C	+ Age + ALT + Creatinine	1.55 (1.44–1.68)	<0.001
Model D	+Age + ALT + Creatinine + Albumin + Hemoglobin	1.46 (1.35–1.58)	<0.001

**Figure 5:**
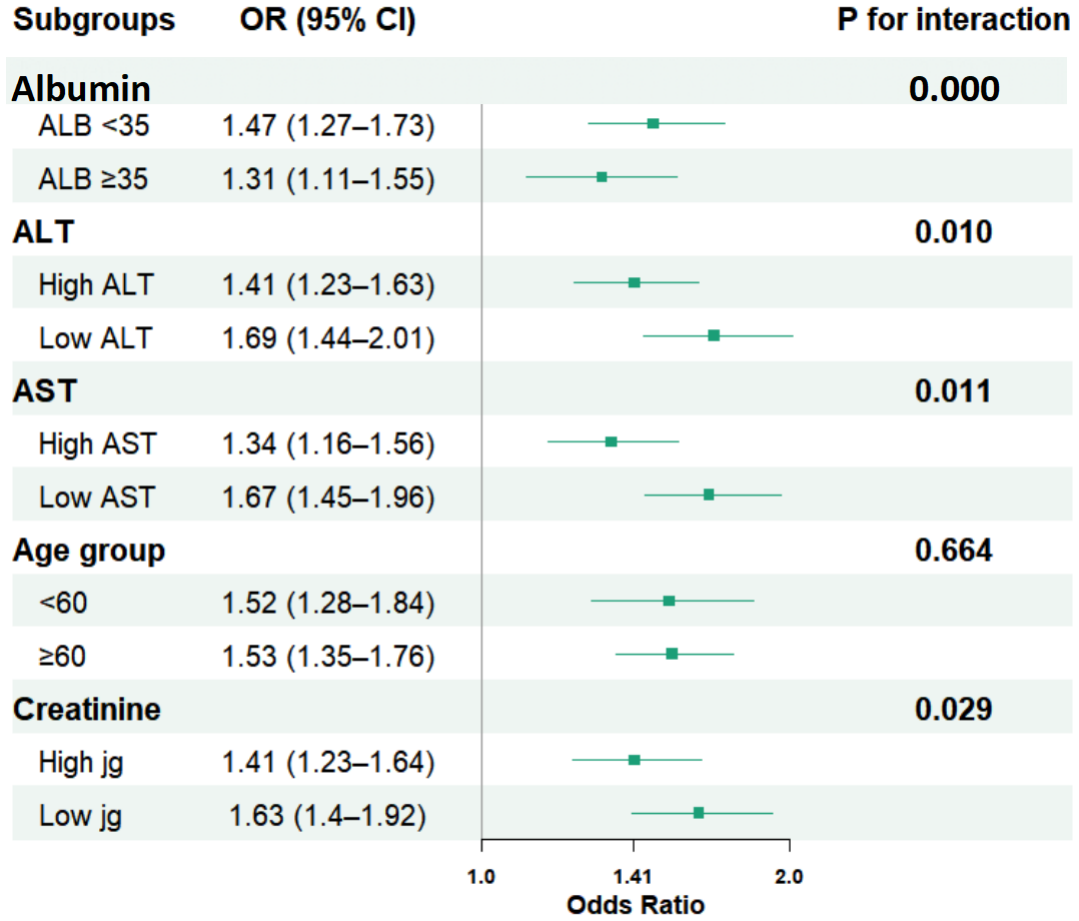
Subgroup analyses of the association between SII and ACN. Forest plot showing odds ratios (ORs) and 95% confidence intervals (CIs) across predefined subgroups. SII was consistently associated with increased ACN risk (all ORs > 1), with significant interactions observed for ALB, ALT, AST, and creatinine, but not for age.

**Figure 5** illustrates that in stratified analyses, SII was linked to a heightened risk in every subgroup, with all odds ratios exceeding 1. While albumin, ALT, AST, and creatinine had significant interactions, age did not. The association appeared stronger in subgroups with lower ALT, AST, and creatinine levels. Taken together, the results of sensitivity and stratified analyses demonstrate that SII is not only an independent and robust predictor but also exhibits context-dependent effects influenced by host physiological status. These observations point out the connection between systemic inflammation and host factors in the progression of colorectal tumors.

### 3.5 Development and assessment of machine learning models

**Figure 6:**
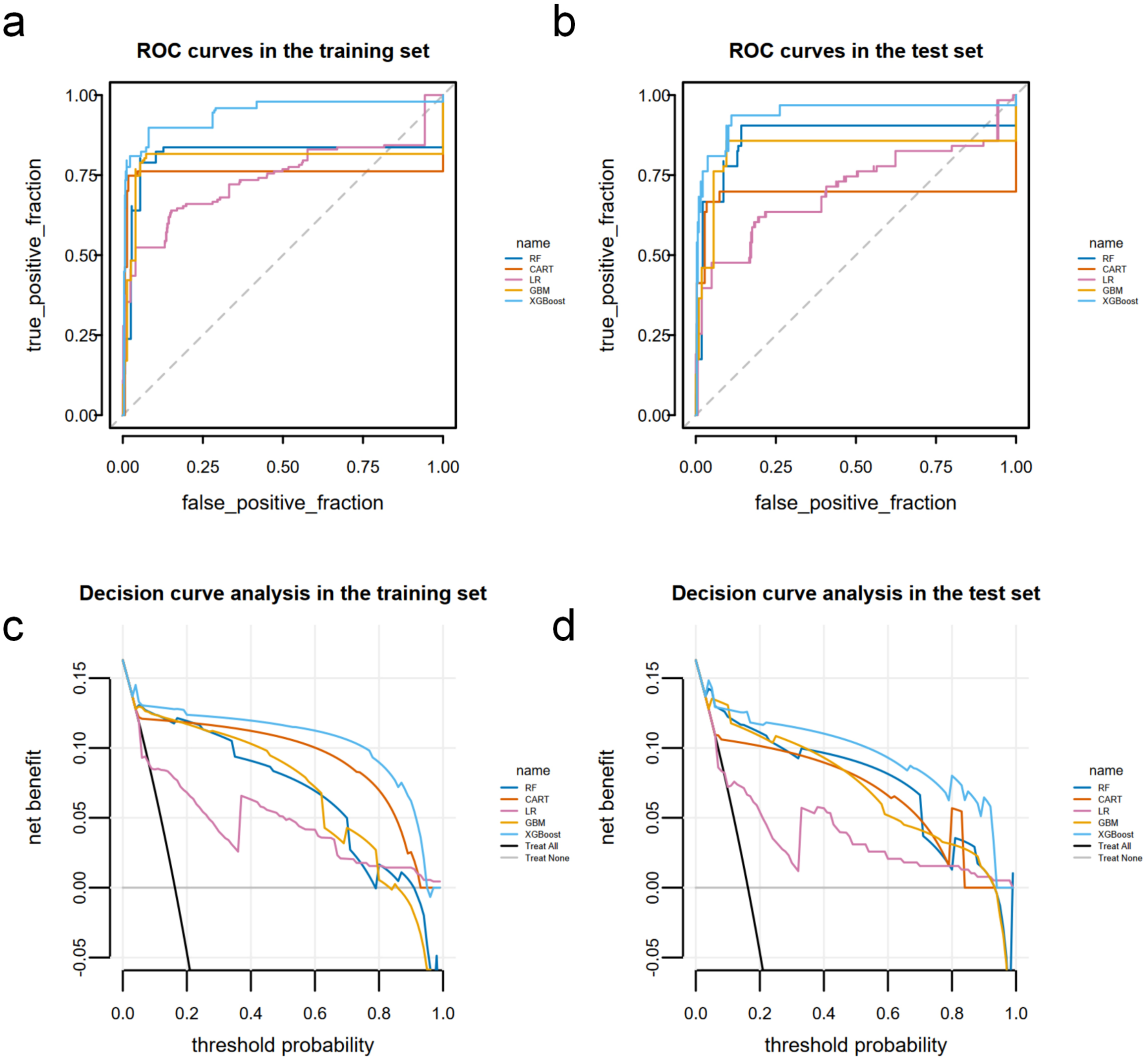
Performance comparison of machine learning models in the training and test sets. **(a)** ROC curves in the training set; **(b)** ROC curves in the test set; **(c)** DCA in the training set; **(d)** DCA in the test set. ROC curves illustrate model discrimination, while DCA evaluates the clinical net benefit across a range of threshold probabilities.

Model performance was examined using ten-fold cross-validation with resampling. The area under the receiver operating characteristic curve (AUC) was used to measure discriminative ability. In the training set, the AUC values (95% CI) for RF, CART, LR, GBM, and XGBoost were 0.889 (0.856–0.923), 0.870 (0.834–0.906), 0.745 (0.688–0.801), 0.885 (0.852–0.919), and 0.956 (0.936–0.976), respectively. In the test group, the corresponding AUC values were 0.918 (0.875–0.960), 0.831 (0.771–0.892), 0.714 (0.626–0.802), 0.900 (0.852–0.947), and 0.955 (0.923–0.987).The performance details of the five machine learning models in both the training and test sets are outlined in **Table 3**, featuring accuracy, sensitivity, specificity, precision, Kappa value, MCC, F1 score, and Brier score. In the training set, the MCC values for RF, CART, LR, GBM, and XGBoost were 0.726, 0.787, 0.429, 0.695, and 0.736, correspondingly. In the test cohort, the MCC values were 0.651, 0.680, 0.355, 0.673, and 0.708. The F1 scores in the training set were 0.771, 0.815, 0.530, 0.745, and 0.777, while in the test set they were 0.709, 0.724, 0.472, 0.725, and 0.747. The Brier scores in the training set were 0.112, 0.051, 0.136, 0.122, and 0.120 for RF, CART, LR, GBM, and XGBoost, respectively. In the test set, the Brier scores were 0.109, 0.073, 0.136, 0.121, and 0.124. Decision curve analysis (DCA) indicated that all models offered clinical advantages over a broad spectrum of threshold probabilities in both the training and test sets, with the curves positioned between the ‘treat-none’ and ‘treat-all’ strategies. Overall, based on multiple performance measures, XGBoost showed the best discriminative ability among the five machine learning models, as shown in **Figure 6**.

**Table 3:** Performance comparison of five machine learning models for predicting advanced colorectal neoplasia in the training and test cohorts.

Dataset	Model	AUC	ACC	SEN	SPE	Precision	F1	Kappa	Brier	MCC
Train	RF	0.889	0.923	0.803	0.946	0.742	0.771	0.725	0.112	0.726
	CART	0.870	0.945	0.748	0.983	0.894	0.815	0.783	0.051	0.787
	LR	0.745	0.815	0.640	0.849	0.452	0.530	0.419	0.136	0.429
	GBM	0.885	0.909	0.816	0.927	0.686	0.745	0.691	0.122	0.695
	XGBoost	0.956	0.916	0.898	0.919	0.684	0.777	0.726	0.120	0.736
Test	RF	0.918	0.894	0.794	0.914	0.641	0.709	0.645	0.109	0.651
	CART	0.831	0.917	0.667	0.966	0.793	0.724	0.676	0.073	0.679
	LR	0.714	0.780	0.603	0.815	0.388	0.472	0.342	0.136	0.355
	GBM	0.900	0.894	0.857	0.901	0.628	0.725	0.661	0.121	0.673
	XGBoost	0.955	0.897	0.937	0.889	0.621	0.747	0.685	0.124	0.708

### 3.6 SHAP-based model interpretability analysis

**Figure 7:**
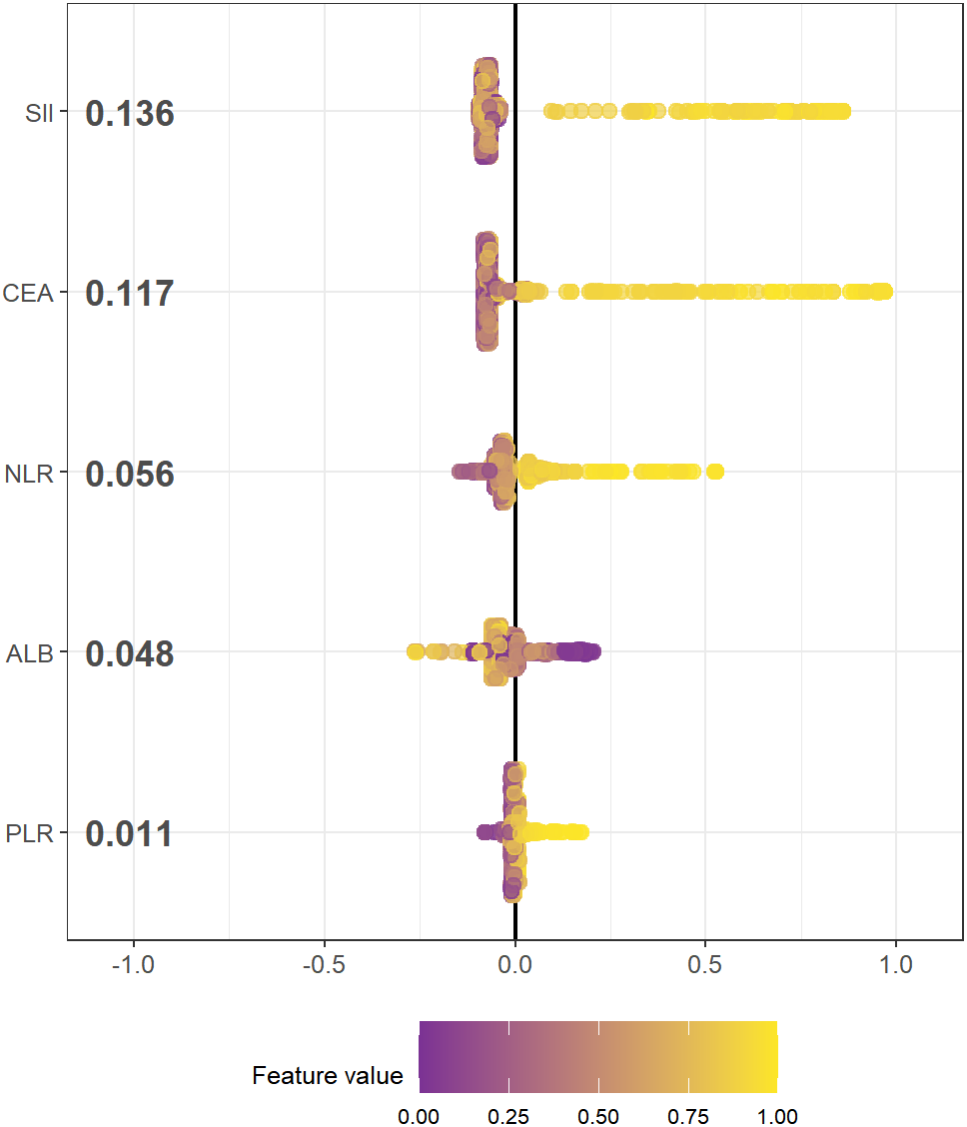
SHAP summary plot for the XGBoost model. The plot illustrates the overall impact of each feature on the model output. Each dot represents an individual patient. Feature values are encoded by color, ranging from purple (low values) to yellow (high values). The x-axis represents SHAP values, where positive values indicate an increased predicted risk of ACN, and negative values indicate a decreased risk. The distribution of points reflects both the magnitude and direction of each feature’s contribution across the study population.

**Figure 8:**
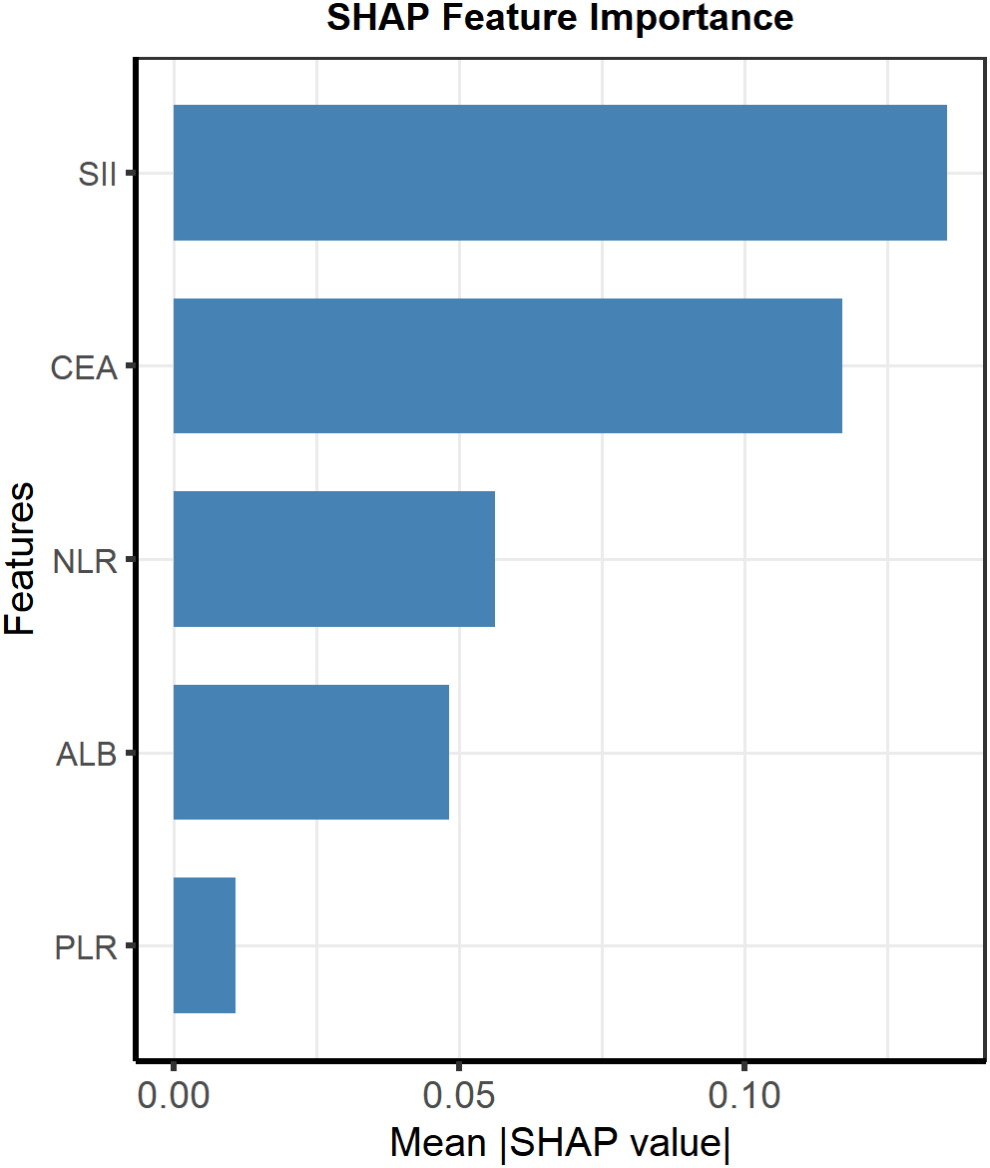
SHAP feature importance ranking for the XGBoost model. Features are ranked according to their mean absolute SHAP values, indicating their relative importance in the model. SII was identified as the most influential predictor, followed by CEA, NLR, PLR, and ALB.

SHAP analysis was used to improve the clinical interpretability of the model by measuring the size and direction of each predictor’s impact on the model’s output, allowing for interpretation on both global and individual scales. At the global level, the SHAP summary plot (**Figure 7**) demonstrated clear directional effects of all variables on risk. Among them, SII emerged as the most influential predictor (**Figure 8**), with higher values consistently associated with positive SHAP values, indicating increased risk, while lower values corresponded to reduced risk. CEA、NLR and PLR also exhibited a stable positive effect, with its contribution becoming more pronounced at higher levels. In contrast, ALB displayed a stable negative association, suggesting that better nutritional status and lower systemic inflammatory burden may exert protective effects.

**Figure 9:**
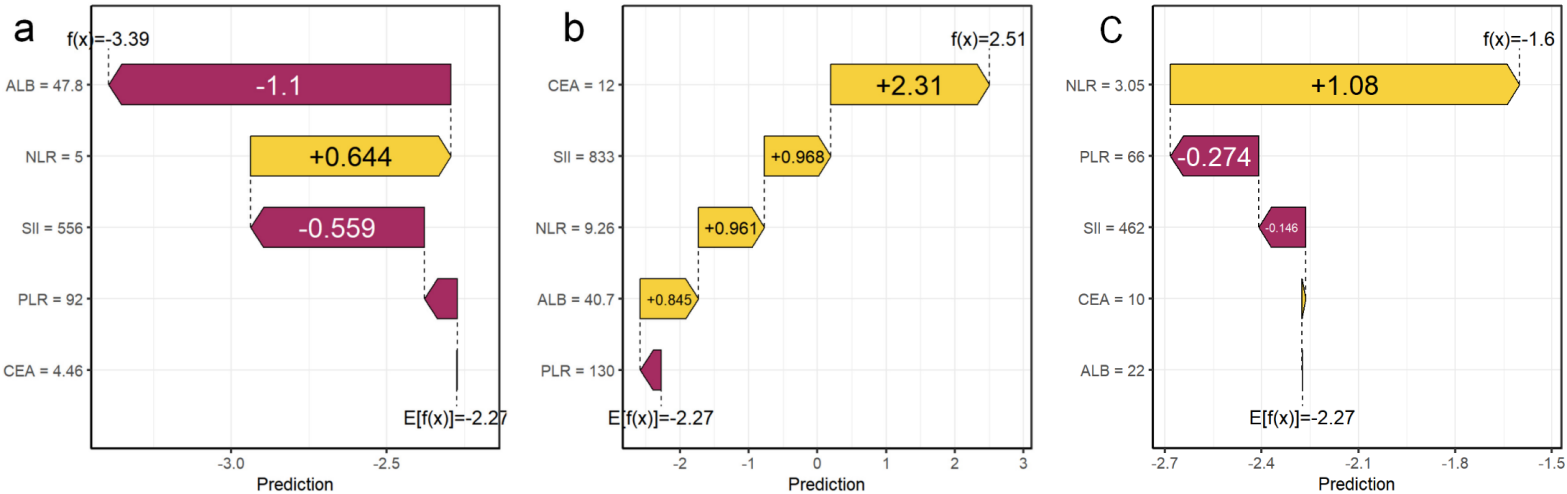
SHAP waterfall plots for individual prediction interpretation. **(a)** A representative patient with ACN; **(b)** a representative patient without ACN. **(c)** a borderline-risk patient. The plots illustrate how each feature contributes to the final prediction, starting from the baseline value to the individual predicted risk.

**Figure 10:**
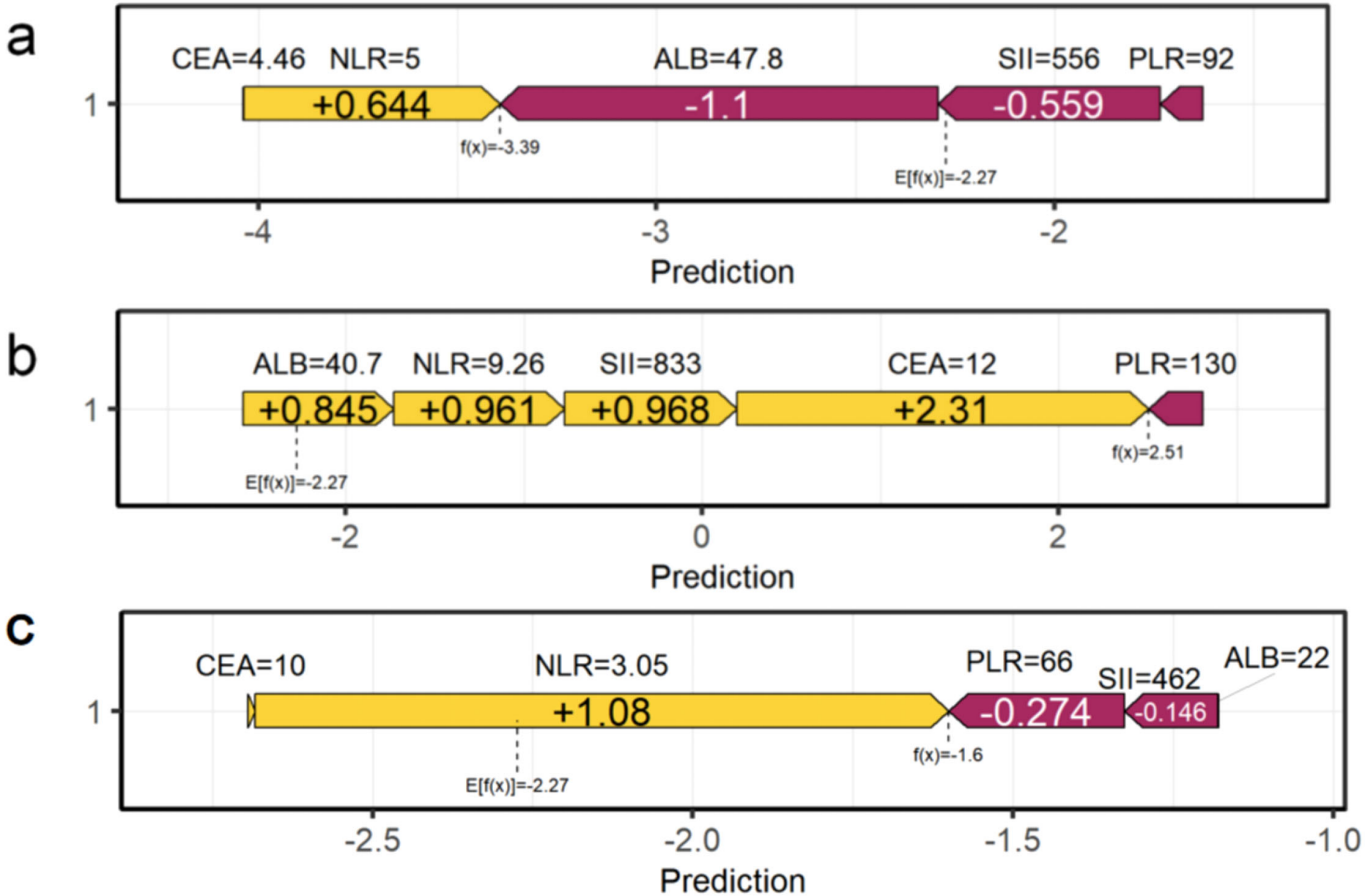
SHAP force plots for individual-level prediction explanation. **(a)** A patient with ACN; **(b)** a patient without ACN. **(c)** a borderline-risk patient. The force plots visualize the direction and magnitude of each feature’s contribution to the prediction.

**Figure 11:**
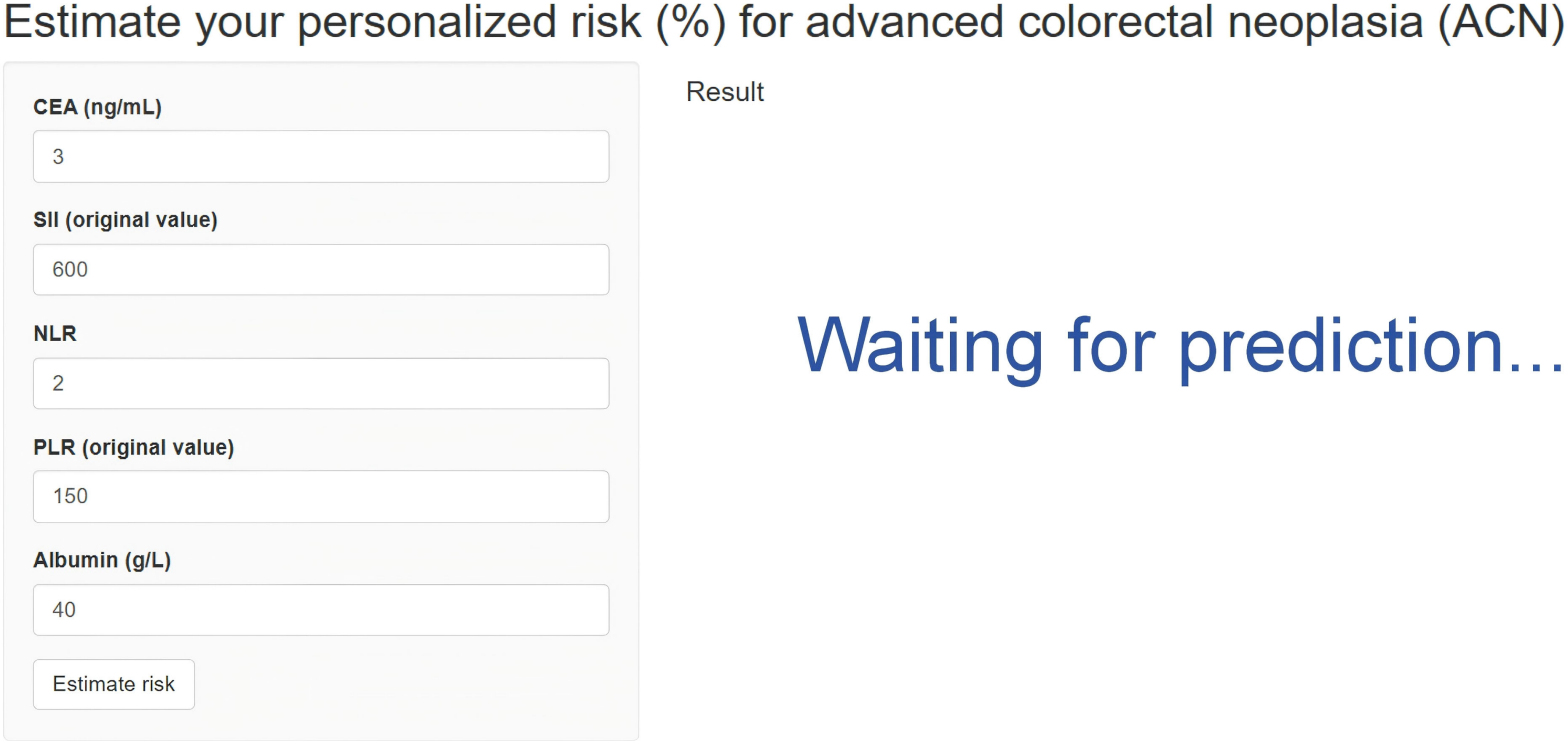
Web-based risk calculator for advanced colorectal neoplasia. Interface of the online prediction tool developed based on the XGBoost model. Users can input relevant clinical variables to obtain individualized risk estimates for advanced colorectal neoplasia.

At the individual level, SHAP waterfall and force plots (**Figures 9**, **10**) illustrated how predictions evolve from the baseline expectation to the final individual output. Representative cases were selected to illustrate typical model behavior. In a representative ACN case (**Figures 9a**, **10a**), the model output increased substantially from the baseline, indicating a high predicted risk. Elevated CEA (12 ng/mL) was the primary driver, followed by increased SII and NLR, while higher PLR and lower ALB further contributed to risk elevation. The combined effect of multiple inflammatory markers and tumor-related indicators resulted in a markedly increased prediction. In contrast, in a non-ACN case (**Figures 9b**, **10b**), the prediction decreased markedly from the baseline, indicating very low risk. In this individual, a high ALB level (47.8 g/L) exerted a strong negative contribution, whereas relatively low SII and CEA had minimal impact on increasing risk. This suggests that favorable nutritional status, together with a low inflammatory burden, can substantially reduce predicted risk. Furthermore, a borderline-risk case (**Figures 9c**, **10c**) demonstrated the model’s ability to balance competing factors. In this example, NLR was the primary contributor to increased risk, whereas PLR, SII, and ALB exerted opposing effects, partially offsetting the risk elevation. As a result, the final prediction remained close to the baseline, reflecting an intermediate-risk profile. To facilitate clinical application, we developed an online web-based calculator based on the final model (https://changchangzhang2001.shinyapps.io/crconline/, **Figure 11**), enabling real-time estimation of individualized risk and supporting clinical decision-making in colorectal cancer assessment.

## 4 Discussion

We created and confirmed a machine learning model using commonly available blood biomarkers to predict the risk of ACN, including advanced adenoma and early-stage carcinoma. The XGBoost model, incorporating five key indicators (SII, CEA, NLR, PLR, and ALB), demonstrated superior predictive performance compared with conventional statistical approaches, with strong discrimination, good calibration, and favorable clinical net benefit. An online calculator was further established to facilitate individualized risk assessment and enhance clinical applicability.

The identified predictors are biologically plausible and consistent with existing evidence. Systemic inflammatory markers, including SII, NLR, and PLR, reflect the balance between inflammatory response and immune function, the disruption of which is closely associated with colorectal tumorigenesis and progression at the stage of advanced neoplasia (31–34). The release of cytokines like IL-6 and TNF-α by neutrophils promotes inflammatory responses, leading to chronic inflammation and dysplastic modifications (34). Platelets secrete factors that promote angiogenesis and boost the adhesion and clustering of tumor cells, thus aiding in the formation of new blood vessels and micro-metastases (35, 36). In contrast, lymphocytes mediate immune surveillance, and their reduction reflects impaired anti-tumor immunity (37, 38). Accordingly, an elevated NLR reflects enhanced neutrophil-driven inflammation and weakened lymphocyte-mediated immune response, while a higher PLR suggests increased platelet-associated pro-tumor activity. SII combines neutrophils, lymphocytes, and platelets to offer a more complete assessment of systemic inflammation and immunosuppression, with its rise being closely linked to a higher risk of ACN.

Serum albumin reflects the balance between hepatic protein synthesis and systemic catabolism. In individuals at high risk of ACN, chronic low-grade inflammation may suppress albumin synthesis via pathways such as IL-6/STAT3 while promoting protein degradation, leading to hypoalbuminemia. In the present study, lower albumin levels observed in the ACN group likely reflect inflammation-related metabolic stress. In contrast, relatively preserved albumin levels may indicate a more favorable physiological state through maintaining oncotic pressure, transporting bioactive molecules, and exerting antioxidant effects (39, 40).

CEA, a tumor-associated protein, is involved in cell adhesion and neoplastic progression. High CEA levels are often linked to a larger tumor load, lower differentiation, and higher invasiveness, and they might suggest a transition from adenoma to high-grade dysplasia or early cancer (41, 42). In this study, integrating CEA with inflammation- and nutrition-related markers enabled the combination of tumor-specific and host-related information, thereby improving the identification of individuals at high risk of ACN. Recent studies have highlighted the prognostic value of inflammation–nutrition composite indices in colorectal cancer (23–25). Extending these findings, our model demonstrates that routinely available biomarkers can capture this axis for ACN risk prediction, supporting their potential clinical utility.

When developing prediction models based on inflammation-related biomarkers, a key concern is the potential influence of confounding and effect modification by variables such as age and renal function. In this study, we systematically addressed these issues through both variable selection and *post-hoc* robustness analyses. Although age and creatinine were statistically significant in univariate analysis, they were not retained in the final multivariable model, as their effects were largely mediated by or collinear with the selected inflammatory and nutritional markers. Sensitivity analyses further confirmed that inclusion of these variables did not alter the significance of the core predictors, suggesting that their effects were not independent. We also explicitly evaluated potential interaction effects. The association between SII and advanced colorectal neoplasia remained stable after sequential adjustment for key clinical variables, with only minimal attenuation, supporting its robustness. Subgroup analyses revealed significant interactions with albumin, ALT, AST, and creatinine, but not with age. Notably, the stronger effect of SII in patients with preserved organ function suggests that baseline inflammation related to organ dysfunction may attenuate its incremental predictive value. In addition, the interaction with albumin supports a synergistic role of nutritional status and systemic inflammation in colorectal tumorigenesis. Collectively, these findings indicate that the selected predictors are robust and largely independent, with limited confounding and biologically plausible effect modification.

Although traditional logistic regression is widely used in clinical prediction models, our results showed that the XGBoost algorithm achieved superior predictive performance, likely due to its ability to capture complex nonlinear relationships and interactions among variables. Consistently, restricted cubic spline (RCS) analysis demonstrated clear nonlinear associations between these indicators and the risk of advanced colorectal neoplasia, which are difficult to model using conventional linear approaches. The observed performance differences between XGBoost and logistic regression (e.g., F1 score: 0.777 vs. 0.530 in the training set and 0.747 vs. 0.472 in the test set) may raise concerns regarding potential overfitting. However, several findings suggest that overfitting is unlikely to be substantial. The minimal difference in AUC between the training and test sets, together with comparable Brier scores, indicates good model stability and calibration. The larger differences in precision and F1 score may partly reflect class imbalance (ACN prevalence of 16.3%), to which tree-based models are more sensitive. To further reduce the risk of overfitting, multiple strategies were implemented. The XGBoost model incorporates built-in L1 and L2 regularization, with hyperparameters optimized using repeated 10-fold cross-validation and early stopping. In addition, the nonlinear patterns identified by the model were consistent with the RCS analysis, supporting the biological plausibility of the findings. The limited number of predictors included in the model may have further contributed to improved model robustness. Nevertheless, additional validation is warranted to enhance generalizability. Future studies should incorporate external validation in independent cohorts and multicenter datasets to provide a more rigorous assessment of model performance.

This study’s notable strength lies in using SHAP to address the ‘black box’ limitation associated with machine learning models (43, 44). Through SHAP summary plots and dependence analyses, we achieved global interpretability of the model, identifying SII and CEA as key contributors to risk prediction. This provides intuitive evidence for clinicians to recognize high-risk indicators at the population level. More importantly, individual-level SHAP visualizations, including force plots, allow case-specific interpretation of predictions (45). In a patient considered high risk for ACN, the primary contributors to the predicted risk were markedly elevated SII and increased CEA, with a reduction in ALB further exacerbating this risk. Model transparency is increased by its dual interpretability on both a global and individual scale, which also supports its application in clinical settings.

To further contextualize the clinical utility of our model, we compared its performance with previous machine learning studies and commonly used noninvasive screening strategies. A recent XGBoost-based model developed by Song et al. (46) reported a sensitivity of 70.8% and a specificity of 83.4% for detecting advanced adenomas. In comparison, our model achieved higher sensitivity and comparable specificity for identifying advanced colorectal neoplasia in the independent test set, suggesting that the integration of inflammatory and nutritional biomarkers may enhance detection performance. Among widely used screening approaches, fecal immunochemical testing (FIT) demonstrates a sensitivity of approximately 20%–50% for advanced precancerous lesions, with specificity ranging from 90% to 95%. Multitarget stool DNA testing improves sensitivity; however, detection rates for advanced adenomas generally remain within 40%–60%. Although colonoscopy remains the gold standard due to its high diagnostic accuracy and therapeutic capability, its invasiveness, cost, and limited patient adherence restrict its widespread use as a primary screening tool. In contrast, our model, based on routinely available blood biomarkers, provides a noninvasive and convenient approach for pre-colonoscopy risk stratification, with the potential to optimize resource allocation and improve screening efficiency. Although the XGBoost model did not demonstrate the best calibration performance among all models, it consistently achieved superior overall performance when considering discrimination, stability, and clinical utility. In particular, it showed the highest AUC values in both the training and test sets, while maintaining comparable calibration and favorable net clinical benefit. Taken together, these findings suggest that XGBoost provides the most balanced performance across multiple evaluation dimensions, supporting its selection as the final model.

To facilitate clinical application, we developed a web-based risk calculator using a Shiny framework, designed to support individualized risk assessment for advanced colorectal neoplasia in routine clinical practice. The model is based on five routinely available laboratory parameters (CEA, SII, NLR, PLR, and ALB), all of which can be readily obtained from standard blood tests, enabling a rapid and noninvasive evaluation of patient risk. This tool may be applicable in several clinical scenarios. First, it can serve as a preliminary screening tool in asymptomatic individuals undergoing routine health examinations, helping to identify those at elevated risk who may benefit from colonoscopic evaluation. Second, in resource-limited settings where colonoscopy capacity is constrained, the model may assist in prioritizing high-risk individuals for referral while reducing unnecessary invasive procedures in low-risk populations. Third, for patients with borderline or inconclusive laboratory findings, the calculator provides a quantitative risk estimate that may support clinical decision-making and follow-up strategies. These applications are aligned with the growing emphasis on risk-adapted colorectal cancer screening. A key consideration for clinical implementation is the standardization of laboratory measurements across different healthcare settings. Although the model was developed using data from a single center, the included biomarkers are derived from widely standardized assays. Nevertheless, variability between institutions may still influence absolute values. Therefore, we recommend interpreting model outputs in conjunction with local laboratory reference ranges and, where appropriate, performing recalibration based on institution-specific data distributions. In terms of workflow integration, the current version of the calculator requires manual data entry via an online interface. Future iterations may incorporate application programming interfaces (APIs) to enable direct integration with electronic health record systems, thereby reducing input errors and improving efficiency. In addition, external validation using multicenter datasets with diverse laboratory platforms will be essential to further evaluate the robustness and generalizability of the tool in real-world clinical settings.

This study has three major strengths. First, it focuses on ACN, a clinically important and potentially reversible stage in the adenoma–carcinoma sequence, rather than established colorectal cancer, thereby providing a more meaningful window for early risk stratification and intervention. Second, the model integrates routinely available biomarkers from complementary biological domains, including tumor-related, inflammatory, and nutritional indicators, allowing a more comprehensive assessment of ACN risk than single-marker approaches. Third, the combination of XGBoost modeling, SHAP-based interpretability, and an online risk calculator enhances both predictive performance and clinical usability, providing an interpretable and practical tool for individualized noninvasive screening.

This study has several limitations that should be considered. First, as a retrospective single-center study, it is subject to potential selection bias and may not fully represent broader populations. Although internal validation was performed using a training–test split and repeated cross-validation, external validation in independent cohorts is still lacking, which may limit the generalizability of the model across different clinical settings. Second, although we systematically assessed confounding and interaction effects through sensitivity and stratified analyses, residual confounding from unmeasured variables cannot be completely excluded. Factors such as lifestyle habits, medication use, and underlying comorbidities may influence both inflammatory status and the risk of advanced colorectal neoplasia, but were not available in the current dataset. Third, significant interactions were identified between SII and albumin, liver function, and renal function. However, these findings were based on subgroup analyses and should be interpreted with caution due to potential sample size limitations within strata. Larger studies are needed to confirm these interactions and further clarify their clinical relevance. Finally, although the model demonstrated strong predictive performance and good interpretability, its clinical applicability requires further validation in prospective and multicenter settings. Our team is currently planning a prospective, multicenter external validation study across three geographically distinct hospitals in Anhui Province, with an estimated enrollment of approximately 800 patients, to assess model transportability and, if necessary, perform site-specific recalibration.

## 5 Conclusion

In this research, we created and confirmed a screening model for advanced colorectal neoplasia using tumor markers and inflammatory indices. The model showed strong predictive capabilities and could be a useful tool for clinicians to identify high-risk individuals early. An internet-based tool for risk calculation was established to enhance individualized risk assessment and clinical decision support.

## Data Availability

The raw data supporting the conclusions of this article will be made available by the authors, without undue reservation.
