# Supplementary material for: A screening model for advanced colorectal neoplasia based on tumor markers and inflammatory indices: a retrospective study with an online risk calculator
Source: Front Oncol. 2026 Jun 22;16:1841258. doi: 10.3389/fonc.2026.1841258 (PMC13333446; doi:10.3389/fonc.2026.1841258)
Supplement: Supplementary file 1 [file DataSheet1.docx]

Supplementary Material

## Supplementary Table

**Table S1. Missing data summary of variables**

| Variable | Missing(n) | Missing(%) |
| --- | --- | --- |
| Age | 16 | 1.24% |
| Ca | 5 | 0.39% |
| Urea | 0 | 0% |
| Creatinine | 9 | 0.70% |
| FIB | 0 | 0% |
| AFP | 0 | 0% |
| CEA | 10 | 0.78% |
| CA199 | 6 | 0.47% |
| CA125 | 0 | 0% |
| CA153 | 0 | 0% |
| CA724 | 0 | 0% |
| ALT | 0 | 0% |
| AST | 0 | 0% |
| TP | 0 | 0% |
| ALB | 0 | 0% |
| A/G | 0 | 0% |
| RBC | 28 | 2.17% |
| Hb | 7 | 0.54% |
| MCHC | 0 | 0% |

**Table S2.Baseline characteristics of patients in the training and test cohorts**

| Variables | Test set(n=387) | Train set(n=903) | P-value |
| --- | --- | --- | --- |
| Age[M(Q1-Q3),years] | 60.00 [53.00, 69.00] | 59.00 [52.50, 69.00] | 0.291 |
| Ca[M(Q1-Q3),mmol/L] | 2.33 [2.00, 2.43] | 2.33 [2.00, 2.42] | 0.588 |
| Urea[M(Q1-Q3),mmol/L] | 5.80 [4.70, 6.90] | 5.70 [4.85, 6.80] | 0.953 |
| Creatinine[M(Q1-Q3),μmol/L] | 68.00 [57.00, 82.00] | 69.00 [58.00, 82.60] | 0.812 |
| FIB[M(Q1-Q3),g/L] | 2.78 [2.38, 3.26] | 2.75 [2.36, 3.21] | 0.424 |
| AFP[M(Q1-Q3),ng/mL] | 2.52 [1.77, 3.45] | 2.46 [1.77, 3.34] | 0.538 |
| CEA[M(Q1-Q3),ng/mL] | 2.56 [1.54, 4.64] | 2.53 [1.63, 4.88] | 0.775 |
| CA199[M(Q1-Q3),U/mL] | 4.98 [2.24, 11.50] | 4.74 [2.12, 9.83] | 0.489 |
| CA125[M(Q1-Q3),U/mL] | 11.70 [8.05, 16.35] | 11.40 [8.30, 16.10] | 0.530 |
| CA153[M(Q1-Q3),U/mL] | 8.10 [6.00, 11.95] | 8.20 [6.30, 11.90] | 0.484 |
| CA724[M(Q1-Q3),U/mL] | 1.87 [1.10, 3.32] | 1.83 [1.00, 3.24] | 0.559 |
| ALT[M(Q1-Q3),U/L] | 21.00 [16.00, 32.00] | 22.00 [16.00, 31.00] | 0.882 |
| AST[M(Q1-Q3),U/L] | 23.00 [18.50, 28.00] | 23.00 [19.00, 29.00] | 0.256 |
| TP[M(Q1-Q3),g/L] | 71.20 [66.75, 75.15] | 71.00 [66.15, 74.75] | 0.292 |
| ALB[M(Q1-Q3),g/L] | 44.80 [38.00, 47.50] | 44.30 [36.90, 47.20] | 0.141 |
| A/G | 1.73 [1.53, 1.91] | 1.71 [1.48, 1.91] | 0.160 |
| RBC[M(Q1-Q3),×10¹²/L] | 4.61 [4.12, 4.99] | 4.57 [4.16, 4.98] | 0.508 |
| Hb[M(Q1-Q3),g/L] | 133.00 [114.50, 145.00] | 133.00 [115.00, 145.00] | 0.994 |
| MCHC[M(Q1-Q3),g/L] | 333.00 [326.00, 340.00] | 333.00 [326.00, 341.00] | 0.642 |
| NLR[M(Q1-Q3)] | 2.16 [1.67, 5.00] | 2.14 [1.66, 4.96] | 0.360 |
| PLR[M(Q1-Q3)] | 120.09 [92.02, 159.14] | 119.18 [88.87, 157.59] | 0.640 |
| PNI[M(Q1-Q3)] | 53.55 [37.80, 57.90] | 53.15 [38.75, 57.38] | 0.773 |
| SII[M(Q1-Q3)] | 392.47 [223.16, 549.39] | 357.76 [206.21, 549.42] | 0.310 |
| FPR[M(Q1-Q3)] | 16.00 [12.00, 23.00] | 17.00 [12.00, 23.00] | 0.489 |
| HRR[M(Q1-Q3)] | 9.00 [8.00, 10.93] | 9.00 [8.00, 10.94] | 0.922 |
| HPR[M(Q1-Q3)] | 0.76 [0.60, 0.90] | 0.75 [0.62, 0.93] | 0.707 |
| LMR[M(Q1-Q3)] | 4.33 [2.90, 5.62] | 4.28 [2.87, 5.74] | 0.928 |
| MLR[M(Q1-Q3)] | 0.20 [0.17, 0.26] | 0.20 [0.16, 0.27] | 0.704 |

**Table S3. Sensitivity analysis for the association between CEA and advanced colorectal neoplasia with sequential adjustment for potential confounders.**

| Model | Adjustments | OR (95% CI) | P value |
| --- | --- | --- | --- |
| Model A | None | 1.27 (1.23–1.32) | <0.001 |
| Model B | + Age | 1.27 (1.23–1.32) | <0.001 |
| Model C | + Age + ALT + Creatinine | 1.27 (1.23–1.32) | <0.001 |
| Model D | +Age + ALT + Creatinine + Albumin + Hemoglobin | 1.19 (1.15–1.24) | <0.001 |

**Supplementary Figure**

**
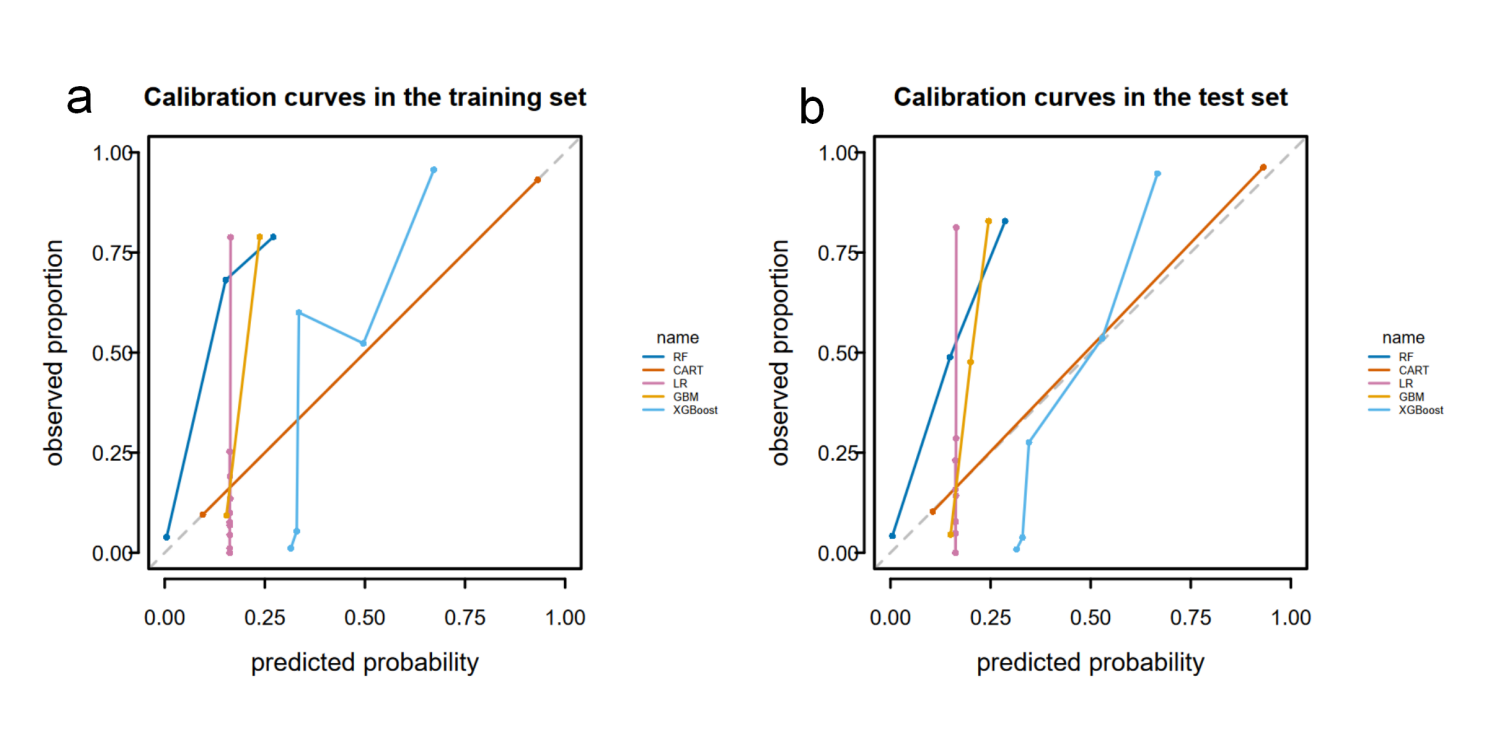
FigureS1. Calibration curves of machine learning models in the training and test sets.**

The x-axis represents predicted probabilities, and the y-axis represents observed event rates. The diagonal dashed line indicates perfect calibration. Curves closer to the reference line reflect better agreement between predicted and observed outcomes, whereas deviations suggest potential overestimation or underestimation of risk.
